# Thyroid cancer overdiagnosis and overtreatment: a cross- sectional study at a thyroid cancer referral center in Ecuador

**DOI:** 10.1186/s12885-020-07735-y

**Published:** 2021-01-08

**Authors:** Paola Solis-Pazmino, Jorge Salazar-Vega, Eddy Lincango-Naranjo, Cristhian Garcia, Gabriela Jaramillo Koupermann, Esteban Ortiz-Prado, Tannya Ledesma, Tatiana Rojas, Benjamin Alvarado-Mafla, Cesar Carcamo, Oscar J. Ponce, Juan P. Brito

**Affiliations:** 1 Otolaryngology Head and Neck Department, Stanford University, Palo Alto, California USA; 2 Knowledge and Evaluation Research Unit, Mayo Clinic, Rochester, MN USA; 3 CaTaLiNa (Thyroid Cancer in LatinAmerica), Quito, Ecuador; 4 Endocrinology Department, Hospital de Especialidades Eugenio Espejo, Quito, Ecuador; 5 One Health Research Group, Universidad de las Americas, Quito, Ecuador, 170137 Quito, Ecuador; 6 Universidad Central del Ecuador, Medical School, Quito, Ecuador; 7 Surgery Department, Hospital de Especialidades Eugenio Espejo, Quito, Ecuador; 8 Molecular Biology Department, Hospital de Especialidades Eugenio Espejo, Quito, Ecuador; 9 Universidad San Francisco de Quito, Quito, Ecuador; 10 School of Public Health and Administration, Universidad Peruana Cayetano Heredia, Lima, Peru; 11 Unidad de Conocimiento y Evidencia, Universidad Peruana Cayetano Heredia, Lima, Peru; 12 Division of Endocrinology, Diabetes, Metabolism, and Nutrition, Mayo Clinic, Rochester, MN USA

**Keywords:** Thyroid Cancer, Histopathology, Surgical, Outcome, Ecuador, Latin-America

## Abstract

**Background:**

In contrast to the rapid increase in thyroid cancer incidence, the mortality has remained low and stable over the last decades. In Ecuador, however, thyroid cancer mortality has increased. The objective of this study is to determine possible drivers of high rates of thyroid cancer mortality, through a cross-sectional analysis of all patients attending a thyroid cancer referral center in Ecuador.

**Methods:**

From June 2014 to December 2017, a cross-sectional study was conducted at the Hospital de Especialidades Eugenio Espejo, a regional reference public hospital for endocrine neoplasia in adults in Quito, Ecuador. We identified the mechanism of detection, histopathology and treatment modalities from a patient interview and review of clinical records.

**Results:**

Among 452 patients, 74.8% were young adults and 94.2% (426) were female. 13.7% had a family history of thyroid cancer, and patients’ median tumor size was 2 cm. The incidental finding was 54.2% whereas 45.8% was non-incidental. Thyroid cancer histology reported that 93.3% had papillary thyroid cancer (PTC), 2.7% follicular, 1.5% Hurtle cells, 1.6% medullary, 0.7% poor differentiated, and 0.2% anaplastic carcinoma. The mean MACIS (metastasis, age, completeness, invasion, and size) score was 4.95 (CI 4.15–5.95) with 76.2% of the thyroid cancer patients having MACIS score less than or equal to 6. The very low and low risk of recurrence was 18.1% (79) and 62% (271) respectively. An analysis of 319 patients with non-metastatic thyroid cancer showed that 10.7% (34) of patients had surgical complications. Moreover, around 62.5% (80 from 128 patients with thyroglobulin laboratory results) of TC patients had a stimulated-thyroglobulin value equal or higher than 2 ng/ml. Overall, a poor surgical outcome was present in 35.1% (112) patients. Out of 436 patients with differentiated thyroid carcinoma, 86% (375) received radioactive iodine.

**Conclusion:**

Thyroid cancer histological characteristics and method of diagnosis are like those described in other reports without any evidence of the high frequency of aggressive thyroid cancer histology. However, we observed evidence of overtreatment and poor surgical outcomes that demand additional studies to understand their association with thyroid cancer mortality in Ecuador.

## Background

The incidence of thyroid cancer (TC) has increased over the last three decades in most countries around the globe [1]. In the United States, an analysis of the Surveillance, Epidemiology, and End Results (SEER) between 1975 and 2015 found that TC incidence has increased from 4.9 to 15 per 100,000 people [2]. Similar epidemiological changes have been observed in Central and South America. From 2008 to 2012, TC rates of incidence in these regions increased 8 to 12 times [3]. In Ecuador, the annual incidence fluctuated from 3 to 22 per 100,000 in the last 16 years, with women having higher rates of incidence than men [4].

Thyroid cancer overdiagnosis seems to be the most important driver of thyroid cancer diagnosis, although the contribution of other risk factors (e.g., obesity) to the rise in thyroid cancer incidence is currently being investigated. In contrast with the rapid increase in TC incidence [5–8], worldwide thyroid cancer mortality has remained low and stable over the last decades [9–11]. In Ecuador, however, thyroid cancer incidence and mortality have increased, and the Ecuadorian thyroid cancer mortality rate is one of the highest in the world [4, 9, 10]. The reason for the high thyroid cancer mortality in Ecuador is unknown.

Ideally, a large population-based study examining the thyroid cancer characteristics and treatment trends may help clarify the triggers of the rates of mortality in Ecuador. However, such a study design is not possible with the current TC data infrastructure in Ecuador. Instead, we conducted a cross-sectional analysis of all patients attending a thyroid cancer referral center in Ecuador to determine possible drivers of high rates of thyroid cancer mortality (type of thyroid cancer diagnosis and surgical outcome). This information might help gain insights into what factors could be contributing to thyroid cancer mortality.

## Methods

### Setting and participants

From June 2014 to December 2017, a cross-sectional study was conducted at the Hospital de Especialidades Eugenio Espejo (HEEE), a regional reference public hospital for endocrine neoplasia in adults in Quito, Ecuador. Ecuador is geographically divided into four major natural regions (Coast, Highland, Amazon, and Galapagos Islands). Due to HEEE being located within the Highland region, its patients come mostly from this area. All the patients who were seen for thyroid cancer at HEEE were included, except the patients who did not have the histopathology report. Patients who had initial management (including surgery) outside HEEE were also included.

### Data collection and variables

Two sources of data were used to collect the variables of interest. First, a study coordinator interviewed eligible patients during their first postsurgical appointment at the endocrine clinic. During this process, the study coordinator captured: 1) demographic characteristics such as age, degree of education, region of residence (Coast, Highland, Amazon, or Galapagos Islands), age at diagnosis, and ethnicity; 2) family history of TC; 3) environmental risk factors; 4) methods of diagnosis (incidental or non-incidental findings). Second, study team members reviewed medical records of included patients to extract the following information: 1) thyroid gland functionality (euthyroid, hypothyroidism, or hyperthyroidism), thyroid ultrasound characteristics, and thyroid nodule fine-needle aspiration(FNA) cytologic results based on Bethesda System; 2) surgical characteristics such as type and extension of surgery; 3) thyroid gland histopathological features including tumor size, type, focality, minor or gross local invasion, and cervical lymph node involvement or distant metastases; 4) TC markers measured after thyroidectomy and before radioactive iodine therapy, including thyroid-stimulating hormone (TSH), stimulated thyroglobulin (sTg), inhibited thyroglobulin (iTg), and anti-thyroglobulin antibodies (aTg); 5) surgical characteristics such as type and extension of surgery, and complications (hypocalcemia < 6 months and > 6 months after procedure, recurrent laryngeal nerve injury); and finally 6) the radioactive iodine treatment, its doses, and scan results.

### Data management

Baseline characteristics data were managed as follows: employment and education were classified according to the National Institute of Statistics and Census (INEC) from Ecuador [11], and thyroid surgery settings were grouped as tertiary (hospitals providing specialized TC management) and non-tertiary hospitals. Furthermore, patients were considered to have a family history of TC when first and second generation-degree relatives had the disease. Based on thyroid histopathologic features, patients were diagnosed as medullary or non-medullary TC, the latter being further classified as differentiated (papillary and follicular), poorly differentiated, undifferentiated (anaplastic), or squamous cell carcinoma [12]. The risk of recurrence in differentiated TC was calculated by using the American Thyroid Association (ATA) 2009 risk stratification system, which classifies patients’ risk of recurrence as low, intermediate, or high [13]. Due to the overwhelming increasing incidence of patients with papillary thyroid cancer (PTC) with an intrathyroidal tumor size of < 1 cm, a new category was included to the ATA risk of recurrence calculator: “very low risk” [14]. Furthermore, the risk of mortality in patients with PTC was estimated based on MACIS score (metastasis, age, completeness, invasion, and size) [15]. A cutoff of 6 was employed to group patients as either low (MACIS < 6) or high risk (MACIS ≥6) of mortality.

**Fig. 1:**
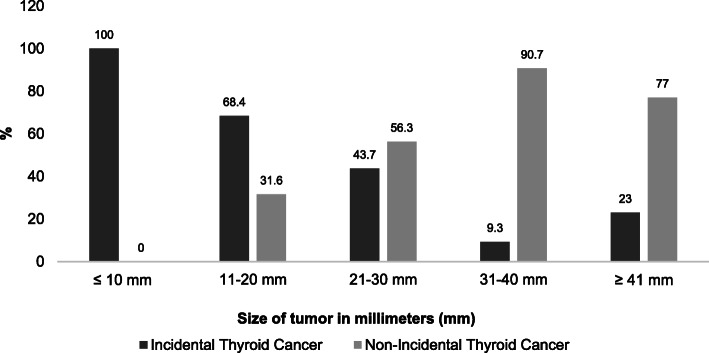
Tumor size by method of diagnosis

Thyroid cancer method of detection was divided in two groups: non-incidental diagnosis (when the TC was found in a symptomatic patient) and incidental diagnosis when a thyroid nodule harboring TC is found during the workup of non-nodular thyroid disease, or during an imaging test requested for reasons unrelated to a thyroid disorder or symptom (e.g., preventive ultrasound), or TC is found incidentally in the histological examination of the thyroid gland removed for a benign condition **(**Fig. 1**)** [16].

We classified the setting of the surgery as either tertiary hospital (HEEE and Hospital SOLCA) or non-tertiary hospital. Moreover, we evaluated the quality of thyroidectomy based on post-operative sTg levels (at least 6 weeks after the procedure) [17–19], and the frequency of surgical complications [20–22]. We considered that the quality of surgery was optimal when there were no post-surgical complications and when patients had a sTg ≤2 ng/dl, and poor when patients had at least one permanent surgical complication or post-operative sTg > 2 ng/dL. Given that surgical complications and post-operative sTg levels could be affected by the presence of metastatic disease, we limited the assessment of the quality of surgical outcomes to patients with non-metastatic differentiated TC undergoing initial thyroid surgery (total thyroidectomy and prophylactic central neck dissection). Before 2016, the criteria for using iodotherapy included the ATA 2009 guidelines; after 2016, the ATA 2015 guidelines were considered.

### Statistical methods

For categorical variables, frequencies and percentages were reported. For numerical variables, we used mean and median with their corresponding standard deviation (SD) or interquartile ranges (IQR), as measurements of central tendency and dispersion. Normal distribution was determined by visual inspection and by using the Kolmogorov-Smirnov test. Our dependent variables used for exploratory analysis were incidental findings and quality of surgery, which are dichotomous variables. For our bivariate and multivariate analysis, we decided to use prevalence ratio (PR) instead of odds ratios (OR) because PR is easier to interpret and OR tend to overestimate the results [23]. To calculate this PR, we planned to use a generalized linear model (GLM) with the binomial family and the *log* link. However, convergence problems were found with some of the variables. Such issues are common [24, 25]. At the end we chose, from all possible solutions, to use Poisson as the family for the GLM with robust variance. For the multivariate analyses, we decided to include in the models for incidental findings and poor quality of surgery all variables in which *p-*value was less than 0.05 and those considered to be important by the investigators. The results are reported as PR and their respective 95% confidence intervals. Statistical analysis was performed with STATA [26].

## Results

From 2014 to 2017, 452 TC patients were included, with 74.8% of the patients between the ages of 20 and 54 years old. The median tumor size of patients was 2 cm [IQ 1.2, 3.1]. Around 94.2% of TC patients were female and 13.7% had a family history of TC **(**Table 1**)**. Thyroid cancer histology was: 93.3% had papillary thyroid cancer (PTC), 2.7% follicular, 1.5% Hurtle cells, 1.6% medullary, 0.7% poor differentiated, and 0.2% anaplastic. The mean MACIS score was 4.95 (IQ 4.15, 5.95) with 76.2% of the TCs having MACIS score equal or less than 6 **(**Table 2**).**

**Table 1:** Characteristics of Thyroid Cancer Patients Before Thyroidectomy

Variable^a^	Total (*n* = 452) n (%)	
Sex		
Female	426	94.2
Males	26	5.8
Age at diagnosis (*mean: 44.6, SD: 14.56)*		
< 20 years old	17	3.8
20–34 years old	106	23.5
35–44 years old	104	23
45–54 years old	128	28.3
55–64 years old	53	11.7
65–74 years old	26	5.8
75–84 years old	16	3.5
> 84 years old	2	0.4
Residence		
Coast	49	10.8
Highland	389	86.1
Amazon	14	3.1
Galapagos	0	0
Employment		
Domestic chores	332	73.5
Student	20	4.4
Labor	100	22.1
Education level		
None	22	4.9
Elementary School	267	59.1
High school	132	29.2
College	31	6.9
Family history of thyroid cancer		
Yes	62	13.7
No	390	86.3
BMI (*n = 298*) (*mean: 28.75, SD: 5.53)*		
Normal	76	25.5
Overweight	113	37.9
Obesity	109	36.6
Self-reported exposure to (*n = 63*)		
Radiation	4	6.3
Chemicals in agriculture	59	93.7
Cigarette Smoking (*n = 393*)		
Yes	17	4.3
No	376	95.7
Thyroid function		
Euthyroid	372	82.3
Hypothyroidism	72	15.9
Hyperthyroidism	8	1.8
Methods of detection		
Non-incidental (palpable nodule)	207	45.8
Incidental	245	54.2
- Ultrasound	229	93.5
- Histology	13	5.3
- Unrelated test	3	1.2
Setting of thyroid surgery (*n = 450*)		
Tertiary Hospital	270	60
Non- tertiary hospital	180	40
Size of tumor (*n = 406)* (*median = 2 cm [IQ 1.2, 3.1])*		
≤ 1 cm	89	21.9
> 1 cm	317	78.1
Focality (*n = 416)*		
Unifocal	230	55.3
Multifocal	186	44.7
Cervical Lymph nodes metastasis (*n = 436)*		
Si	211	48.4
No	225	51.6
MACIS score *(n = 408)* (*median = 4.95 cm [IQ 4.15, 5.95])*		
≤ 6	311	76.2
> 6	97	23.8
Histopathology, *(n = 447)*		
Papillary	417	93.3
Follicular	12	2.7
Hurtle cells	7	1.5
Poor differentiated	3	0.7
Anaplastic	1	0.2
Medullary	7	1.6
Risk recurrence (*n = 437)*		
Very low risk	79	18.1
Low risk	271	62.0
Indeterminate risk	49	11.2
High risk	38	8.7

^a^All variables without a specific number of patients were calculated from the whole population (*n* = 452). All the others show the number of people from which the variables were available

**Table 2:** Factors associated with the prevalence of diagnosis

	Incidental		Univariate analysis		Multivariate analysis	
Variables	No	Yes	PR (95% CI)	p		
	*n* = 206	*n* = 246				
Age, mean (SD)	43.0 (16.1)	46.3 (13.4)	1.01 (1.00,1.01)	0.037	1.01 (1.00, 1.02)	0.033
Sex, n (%)						
Male	14 (6.8)	12 (4.9)	Reference	0.433		
Female	192 (93.2)	234 (95.1)	1.19 (0.78, 1.81)			
Positive Family history n (%)						
No	179 (86.5)	211 (86.1)	Reference	0.914		
Yes	28 (13.6)	34 (13.8)	1.01 (0.79, 1.29)			
BMI, (*n = 299) mean, (SD)*	*n = 142* 28.6 (5.7)	*n = 157* 29.2 (5.8)	1.00 (0.99, 1.02)	0.630		
Tumor size in mm, (*n = 406)* mean, (SD)	*n = 177* 35.7 (17.9)	*n = 229* 22.3 (12.3)	0.96 (0.95, 0.97)	0.000	0.96 (0.94, 0.97)	0.000
Multifocal (*n = 328*), n (%)						
Unifocal, n (%)	103 (55.4)	127 (55.2)	Reference	0.974	Reference	0.000
Multifocal, n (%)	83 (44.6)	103 (44.8)	1.00 (0.84, 1.19)		1.32 (1.13, 1.54)	
Positive Cervical Lymph nodes, n (%)						
No	93 (46.3)	132 (56.2)	Reference	0.041	Reference	0.305
Yes	108 (53.7)	103 (43.8)	0.83 (0.70, 0.99)		1.09 (0.93, 1.27)	
MACIS score (*n = 406)*, mean (SD)	5.6 (1.6)	4.9 (1.4)	0.86 (0.79, 0.92)	0.000	0.92 (0.81, 1.04)	0.184
Risk recurrence *(n = 437)*						
Very low risk	0	79 (33)	Reference^a^			
Low risk	147 (73.5)	124 (52.3)				
Intermediate risk	33 (16.5)	16 (6.8)	0.56 (0.37, 0.85)	0.006	0.98 (0.67, 1.43)	0.902
High risk	20 (10.0)	18 (7.6)	0.77 (0.54, 1.11)	0.163	1.04 (0.82, 2.16)	0.239

^a^ For univariate and multivariate analysis low and very low risk categories were combined and taken as reference

### Mechanism of detection

**Fig. 2:**
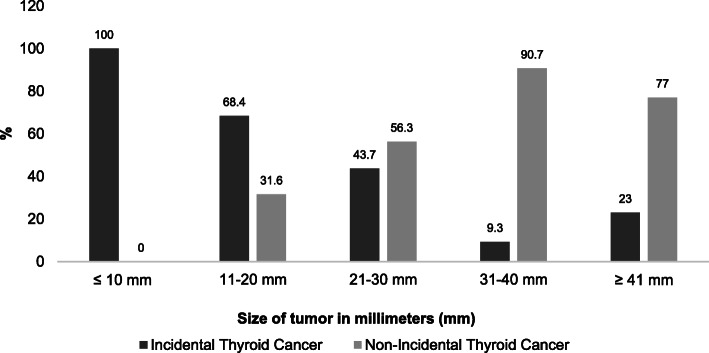
Tumor size by method of diagnosis

The methods of TC diagnosis were: 54.2% incidental (93.5% by ultrasound, 5.3% histology, and 1.2% unrelated test) and 45.8% non-incidental (palpable and symptomatic nodule) **(**Fig. 1**)**. Furthermore, 100% of patients with microcarcinoma (≤10 mm) were incidental finding; the proportions further fell to 68.4, 43.7, 9.3, and 23% for the 11-20 mm, 21-30 mm, 31-40 mm and ≥ 41 mm groups, respectively **(**Fig. 2**).** The univariate analysis showed that age, tumor size, and MACIS score were associated with the mechanism of detection (Table 3).

**Table 3:** Factors associated with poor optimal surgical outcomes

	Univariate analysis		Multivariate analysis	
	PR (95% CI)	***P*** value	PR (95% CI)	***P*** value
**Sex**				
Male	Reference	0.295		
Female	0.74 (0.42, 1.30)			
**Age, mean (SD)**	1.00 (0.99, 1.01)	0.684		
**BMI**	1.02 (0.99, 1.05)	0.191		
**Setting of Surgery**				
- Tertiary Hospital	Reference	0.878	Reference	0.947
- Non- tertiaty hospital	0.98 (0.71, 1.32)		1.01 (0.75, 1.37)	
**Tumor size** ***(n = 306),*** **mean (SD*)***	1.01 (1.00, 1.02)	0.001	1.01 (1.00, 1.02)	0.182
**Tumor focality, n (%)**				
- Unifocal	Reference	0.425		
- Multifocal	1.13 (0.84, 1.53)			
**Positive Central Cervical Lymph nodes metastasis, n (%)**				
- No	Reference	0.004	Reference	0.017
- Yes	1.54 (1.15, 2.06)		1.45 (1.07, 1.97)	
**MACIS score**	1.14 (1.02, 1.28)	0.017	1.08 (0.94, 1.24)	0.285
**Histology variant**				
- **Non- aggresive**	Reference	0.651		
- **Aggresive**	1.11 (0.70, 1.79)			
**Risk recurrence**				
Very low risk	Reference^a^			
Low risk				
Indeterminate risk	0.95 (0.51, 1.77)	0.861		
High risk	0.95 (0.19, 4.73)	0.946		

^a^ For univariate and multivariate analysis low and very low risk categories were combined and taken as reference

### Treatment modalities

#### Surgical characteristics and outcomes

All patients were treated with total thyroidectomy. The operations were performed by the HEEE surgery team (7 surgeons) in 46% (205) of patients. Other patients had operations in other institutions and were transferred to the HEEE to continue the follow-up. The neck dissection was performed as follows: 46% (209) central compartment, 22% (97) lateral compartment, and 32% (146) did not have neck dissection. Of patients with central compartment neck dissection, 16% (34) had more than 5 positive lymph nodes removed. An analysis of 319 patients with non-metastatic differentiated thyroid carcinoma (DTC) showed that 10.7% (34) of patients had surgical complications, 7.8% (25) of patients developed permanent hypoparathyroidism, 2.2% (7) had recurrent laryngeal nerve injury, and 0.6% (2) showed spinal nerve injury. Moreover, around 61% (80 from 128 patients with thyroglobulin laboratory results) of TC patients had a sTg value equal or higher than 2 ng/ml. By using both surgical complications and sTg values, the percentage of patients who had a poor surgical outcome was 35%. The univariate analysis showed that the tumor size, MACIS score and the presence of metastatic cervical lymph nodes in the central compartment were associated with poor surgical outcome. However, in multivariate analysis, only metastatic cervical lymph nodes were associated with poor surgical outcome (PR = 1.45 [IC:1.07, 1.97]) **(**Table 4**).**

**Table 4:** Radioactive Iodine and risk of recurrence

Risk recurrence	Yes ***n*** = 375 (89.3%)	No ***n*** = 45 (10.7%)	PR (95% CI)	***P*** value
**Very low risk (micro PTC) n (%)**	49 (63.6)	28 (36.4)	0.2 (0.13, 0.35)	0.001
**Low risk**	248 (95)	13 (5)		
**Indeterminate risk**	43 (91.5)	4 (8.5)		
**High risk**	35 (100)	0 (0)		

#### Iodine therapy

Out of 436 patients with DTC, 86% (375) received RAI. The median dose of RAI was 100 mCi (IQR: 100–150) and the median lapse between surgery and RAI therapy was 4 months (IQR: 3–7 months). 95% of people with very low risk and low risk received RAI treatment **(**Table 5**).**

**Table 5:** Radioactive Iodine Treatment in patients with thyroid cancer by their risk of recurrence

Variables	Radioactive Iodine Treatment			
	Yes	No	PR (95% CI)	***p*** value
	***n*** = 375 (89.3%)	***n*** = 45 (10.7%)		
*Risk of recurrence*				
- Very low risk (micro PTC) n (%)	49 (63.6)	28 (36.4)	0.2 (0.13, 0.35)	0.001
- Low risk	248 (95)	13 (5)		
- Indeterminate risk	43 (91.5)	4 (8.5)		
- High risk	35 (100)	0 (0)		

## Discussion

We conducted a cross-sectional analysis of all TC patients receiving care at a regional reference hospital in Ecuador. This analysis revealed that 74.8% of TC patients were between 20 and 54 years old, and the majority was papillary thyroid cancer at low or very low risk of recurrence. Approximately half of these cases were found incidentally, and a quarter of TC patients had a poor surgical outcome. Despite being mostly low risk for cancer, all patients received total thyroidectomy, and the majority received RAI.

Although this sample only represents a small subset of all thyroid cancers in Ecuador, histological characteristics and methods of diagnosis are similar to the ones described in other reports [27–31]. We did not see an increased frequency of aggressive thyroid cancer histological findings that might explain the increase in thyroid cancer mortality in Ecuador. We observed that the majority of thyroid cancer cases were of low risk of recurrence and mortality. Moreover, we found that more than half of thyroid cancers were diagnosed incidentally, and the minority of patients presented with symptoms resembling findings in countries where thyroid cancer overdiagnosis drives increasing incident trends [32–35]. The driver of incidental thyroid cancer in this cohort was the used of neck ultrasound. This finding is consistent with other studies that shows that the use of thyroid ultrasound has increased at a rate of 20% per year from 2002 through 2013 in the United States [36, 37] and was associated with more thyroid cancer diagnosis, this cancer found by neck ultrasound was mostly of low risk. Although thyroid cancer histology and mode of presentation did not show any hint to explain thyroid cancer increased mortality, we found that there was evidence of overtreatment and poor surgical outcomes. One-third of patients had either surgical adverse events or a post-surgical Tg value that suggested residual benign or malignant thyroid tissue. Persistent thyroglobulin in our cohort may be the result of both insufficient surgical treatment of cervical lymph node metastases and a significant remnant of thyroid tissue in the glandular bed. The high frequencies of poor surgical outcomes suggest a lack of surgical thyroid cancer expertise [38–40]. In Ecuador, there are no residency programs dedicated to training surgeons about the treatment of TC. The few existing thyroid focused surgeons are insufficient in covering the rising demand for new patients with this tumor; therefore, before 2017 most of our patients underwent a thyroidectomy with a general surgeon and most of the patients underwent prophylactic central or lateral neck dissection (68%). In light of this, a retrospective study assessed the safety and efficiency of thyroid surgery, it found more hypocalcemia < 2.0 mmol/l (32.8 vs. 22.0%) and postoperative hemorrhage (5.6% vs. 1.9%) in surgeries performed by general surgeons [40]. These poor surgical outcomes might be higher among countries affected by higher rates of TC diagnosis that have limited thyroid surgical expertise [21] (e.g., Ecuador). Yet, most TC patients do not receive care or treatment in a reference hospital, and thus, they may be at higher risk of complications (e.g., hypocalcemia, recurrent laryngeal nerve injury, etc.) and perhaps unrecognized death due to thyroid cancer surgery.

Another driver of the increased thyroid cancer mortality in Ecuador, not assessed in this study, may be attribution bias. That is, patients with thyroid cancer who died, and the cause of death is attributed to thyroid cancer even if cancer was likely not the cause of death [41]. This misclassification bias exaggerates cancer-specific mortality. Morticians not familiar with thyroid cancer prognosis may be more willing to allocate cause of death to thyroid cancer when the chain of events leading to death is unclear or unknown. Moreover, we observed that the majority of thyroid cancer cases were of low or very low risk of recurrence, however, most of them received high doses of RAI therapy. In our study, it is not clear if the rise observed in the use of RAI is associated with diagnosis of higher-risk tumors or if clinicians continue to prescribe RAI due to a lack of knowledge or for thyroid remnant ablation. Although RAI use would not have a detrimental impact on thyroid cancer mortality, its use adds to the patient’s burden of treatment and risk of adverse events [42–44].

### Limitations and strengths

This study has several limitations. This is not a population-based study; therefore, selection bias may influence our results. Furthermore, there were patients with missing data, lowering our sample size and confidence in the estimates. Information about the histopathological characteristics and post-surgical treatment were unavailable because not all patients began the treatment in HEEE and some of them came to this hospital after surgery or after radioactive therapy was performed. Moreover, in the interview of the patients, the question of family history was exposed to recall bias. Finally, we were not able to provide information about the outcomes for these patients as this data is currently being collected as data for a subsequent study. Despite these limitations, this study has several strengths. First, patient data was collected by extractors trained in thyroid cancer treatment and diagnosis using a well-designed extraction form. Second, our inclusion criteria and case finding process secured that all patients treated at the hospital were include for analysis. Finally, our team included a multidisplinary team of clinicians, thyroid cancer researched and epidemiologists who contributed to planning, execution, and dissemination of study’s results.

## Conclusion

Considering the paucity of population-based cancer registries in Ecuador, this study provides additional information about the thyroid cancer diagnosis and treatment in a tertiary referral center in Ecuador. We observed that thyroid cancer histological characteristics and methods of diagnosis are like those described in other reports without any evidence of the high frequency of aggressive thyroid cancer histology. However, we observed evidence of overtreatment and poor surgical outcomes that demand additional studies to understand their association with thyroid cancer mortality in Ecuador. This study is significant because it shows the high rate of overtreatment for patients seen in a tertiary care center for thyroid cancer in Ecuador, particularly the high frequency of surgical adverse outcomes among patients with low-risk thyroid cancer, who may benefit from non-surgical options for their management. These results further invite to better understand the potential association of overtreatment and thyroid cancer mortality.

## Data Availability

Since data came from the medical records where sensitive information is collected, no database is publicly available. Nevertheless, anonymized information can be shared privately upon reasonable request at e.ortizprado@gmail.com or paosolis@stanford.edu.

## Abbreviations

TC: Thyroid Cancer
HEEE: Hospital de Especialidades Eugenio Espejo
MACIS: Metastasis, age, completeness, invasion, and size
DTC: Differentiated thyroid carcinoma
RAI: Radioactive iodine
SEER: Surveillance, Epidemiology, and End Results
FNA: Fine-needle aspiration
TSH: Thyroid-stimulating hormone
sTg: Stimulated thyroglobulin
iTg: Inhibited thyroglobulin
aTg: Anti-thyroglobulin antibodies
INEC: National Institute of Statistics and Census
ATA: American Thyroid Association
PTC: Papillary thyroid cancer
SD: Standard deviation
IQR: Interquartile ranges
PR: Prevalence ratio
OR: Odds ratios
GLM: Generalized linear model
